# Prevalence and associated factors of molar incisor hypomineralization in children: a cross-sectional study

**DOI:** 10.1186/s12903-025-07071-2

**Published:** 2025-11-25

**Authors:** Selin Sena Yılmaz, İlhan Uzel, Fahinur Ertuğrul, Şule Gökçe, Emine Burçe Dörtkardeşler, Güneş Ak

**Affiliations:** 1 Department of Pediatric Dentistry, Faculty of Dentistry, Ege University, Izmir, Türkiye; 2 Private Dentist (Practice), Muğla, Türkiye; 3 Department of General Pediatrics, Faculty of Medicine, Ege University, Izmir, Türkiye; 4 Department of Clinical Biochemistry, Faculty of Medicine, Ege University, Izmir, Türkiye

**Keywords:** Molar incisor hypomineralization, Nutrition, Etiology of MIH

## Abstract

**Background:**

Molar Incisor Hypomineralization (MIH) is a qualitative enamel defect affecting first permanent molars often involving incisors. It presents clinical challenges including hypersensitivity, rapid caries, and restorative difficulties. Though its etiology is unclear, systemic and environmental factors have been implicated.

**Objective:**

This study aimed to determine the prevalence and associated factors of MIH and to assess the risk factors.

**Methods:**

A cross-sectional study was conducted on children aged 6–12 years in İzmir, Türkiye. Among 700 children examined, 50 diagnosed with MIH were included in the case group, while 50 healthy children formed the control group. Parental interviews assessed sociodemographic, prenatal, perinatal, postnatal factor. Biochemical parameters were analyzed appropriate statistical tests.

**Results:**

MIH prevalence was 12% and 76% had severe defects (MIH-2), and 24% had mild opacities (MIH-1). Significant associations were observed with socioeconomic status, maternal employment, birth weight, antibiotic use and respiratory infections (*p* < 0.005). Exclusive breastfeeding duration was longer in controls (*p* = 0.020).

**Conclusion:**

MIH is a multifactorial condition influenced by systemic and environmental factors. Early diagnosis and prevention are essential. Longer breastfeeding duration may offer a protective effect. Larger longitudinal studies are needed to better understand associated factor of MIH.

**Supplementary Information:**

The online version contains supplementary material available at 10.1186/s12903-025-07071-2.

## Introduction

Molar Incisor Hypomineralization (MIH) is a developmental enamel defect that primarily affects the first permanent molars and frequently the incisors. Clinically, MIH leads to rapid dental caries, hypersensitivity, aesthetic concerns and compromised restorative outcomes, presenting a significant challenge for pediatric dental care [1]. Despite global recognition of MIH and its increasing prevalence, the etiology remains complex and multifactorial, involving systemic, genetic, and environmental influences during critical stages of tooth development [2].The etiology of MIH is multifactorial, involving systemic, genetic, and environmental factors during the prenatal, perinatal, and postnatal stages [3, 4]. Numerous international studies have investigated MIH prevalence and risk factors; however, there is a limited body of region- spesific evidence from Turkey, particularly concerning the western urban region of İzmir. Previous studies from Turkey have reported varying prevalence rates and heterogeneous risk associations, indicating a need for localized epidemiological data that accounts for sociodemographic, perinatal and systemic health variables [5–7]. Although no clear causal relationship has been established, risk factors include vitamin D deficiency, antibiotic use, childhood infection illnesses, and atopic diseases such as dermatitis and asthma [3, 8–10]. Globally, MIH affects approximately 12.9% of children, with regional variations [11]. In Turkey, its prevalence ranges from 9.1% to 14.2%, with severity classified as mild (limited opacities) or severe (post-eruptive breakdown or restorations) [5–7].

Therefore, this study aims to determine the prevalence of MIH among school-aged children in İzmir amd to identify associated etiological factors, contibuting context-specific insights to the global understanding of MIH.

## Materials and methods

This quantitative, descriptive study was conducted at Ege University Faculty of Dentistry, Department of Pediatric Dentistry, and Faculty of Medicine, Department of Pediatrics, Division of General Pediatrics, between September 2023 and January 2024.

### Ethics

Ethics committee approval was obtained from the Ege University Faculty of Medicine Medical Research Ethics Committee. Informed consent was secured from parents, and assent was obtained from children. This study was conducted in accordance with the principles of the Declaration of Helsinki.

### Study population

Between September 2023 and January 2024, 700 patients visited the Department of Pedodontics at Ege University Faculty of Dentistry for examination, and MIH was diagnosed in 84 of them. Out of these 84 patients, 50 volunteers who met the inclusion criteria and consented to participate were randomly selected for the study. The control group consisted of 6–12-year-old patients with fully erupted molars who had no MIH diagnosis. For the case–control component of the study, children diagnosed with MIH according to the EAPD criteria were assigned to the case group, while children without MIH and other developmental enamel defects were assigned to the control group. All eligible participants within the study period who met the inclusion criteria were invited to participate; no randomisation was performed in the selection of cases or controls.

The MIH examination was conducted in the deparment of pediatric dentistry under natural daylight. This study employed a convenience sampling method, including children who attended the university’s paediatric dentistry clinic. The clinic serves patients from a wide range of socioeconomic backgrounds and, as a tertiary care centre, also receives referrals from other dental practitioners, which increases the diversity of the patient population. Two experienced paediatric dentistry specialists, both fully competent in diagnosing MIH, examined all children. Both examiners were trained in MIH diagnosis and had prior clinical experience in identifying MIH using the European Academy of Paediatric Dentistry (EAPD) criteria.

During the procedure, the children sat upright in a chair. The dentists moistened the teeth and used a dental mirror and probe to eliminate any food debris if necessary. Each tooth (4 first permanent molars, 8 incisors and 4 canines) was assessed based on the EAPD criteria for MIH. For MIH diagnosis, at least one affected first permanent molar must be present and frequently associated with affected incisors.

MIH diagnosis was performed according to the European Academy of Paediatric Dentistry (EAPD) criteria, using the severity grading tool. These criteria were selected because they are widely used in clinical and epidemiological studies and were in routine use in our department at the time the study was designed and initiated. Although the Ghanim et al. (2015) criteria and their training manual (2017) provide a detailed scoring system and training guidance, our department had already adopted the EAPD criteria for clinical use and research purposes. The EAPD criteria were chosen to ensure consistency with previous studies conducted in our institution and to facilitate comparability with other prevalence studies in the literature.

### Questionnaire evaluation

The potential aetiological factors were assessed with a questionnaire comprised of 29 questions and were sent to parents/caretakers in addition to the study consent form. A total of 100 questionnaires were completed and returned. A questionnaire was specifically developed for this study.

A structured questionnaire was developed by the research team to collect data on possible prenatal, perinatal, and postnatal factors that may be associated with MIH. The questions were based on previous literature investigating MIH-associated risk factors and were reviewed by three paediatric dentistry specialists, two pediatrician for content relevance and clarity. A pilot test was conducted with a small group of parents to ensure that the questions were understandable. Although no formal statistical validity and reliability testing was performed, the items were refined based on expert feedback and pilot results.

To reduce recall and response bias, parents were encouraged to refer to written health records such as vaccination cards, birth records, or paediatric medical files when answering questions related to the child’s early life. Additionally, interviews were conducted face-to-face by the examiners to clarify questions when necessary and minimise misunderstandings.

The questionnaire contained following sections: Demographic data (child’s age, gender, place of birth), whether the mother was healthy or taking any medicines during pregnancy, how was the baby delivered, whether there were any complications during or before childbirth and child’s birth weight, average breastfeeding period, child’s medical history of first three years (diarrhea, tonsillitis, pharyngitis, digestive system diseases, atopic diseases, pneumonia, respiratory tract infections, asthma, frequent high fever (39 C), otitis media, chronic renal failure, urinary infections and childhood diseases as a rubella, scarlet fever, chickenpox).

Exclusion criteria included systemic diseases, dental anomalies, orthodontic treatment, and missing permanent teeth. Children with systemic diseases known to directly affect enamel formation (such as amelogenesis imperfecta, chronic kidney disease, or severe metabolic disorders) were excluded to avoid confounding factors that could mask MIH lesions. Similarly, children with developmental dental anomalies unrelated to MIH (such as amelogenesis imperfecta, dentinogenesis imperfecta, or enamel hypoplasia due to local trauma) were excluded to ensure accurate diagnosis. The differentiation between MIH and other enamel defects was made according to the European Academy of Paediatric Dentistry (EAPD) diagnostic criteria, considering lesion distribution, demarcation, and clinical history. Both examiners were paediatric dentistry specialists experienced in distinguishing MIH from other developmental enamel defects.

### Clinical evaluation

**Fig. 1:**
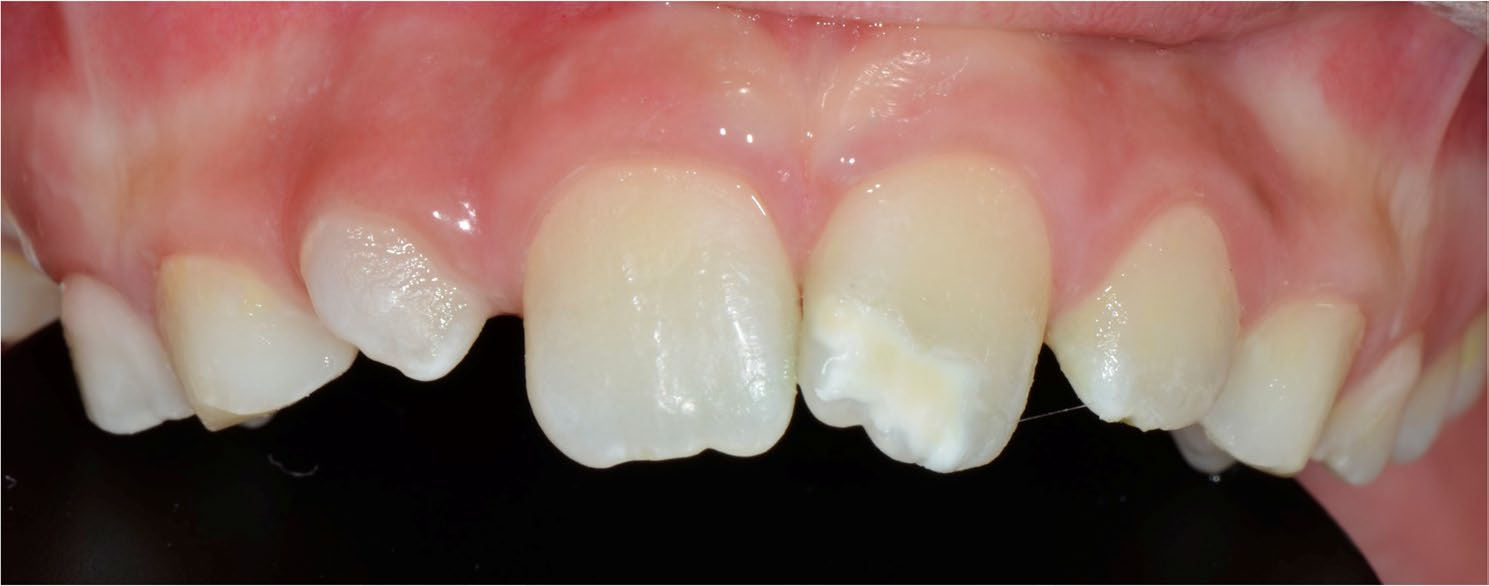
MIH-1: Limited opacities on maxillary central and lateral incisors

**Fig. 2:**
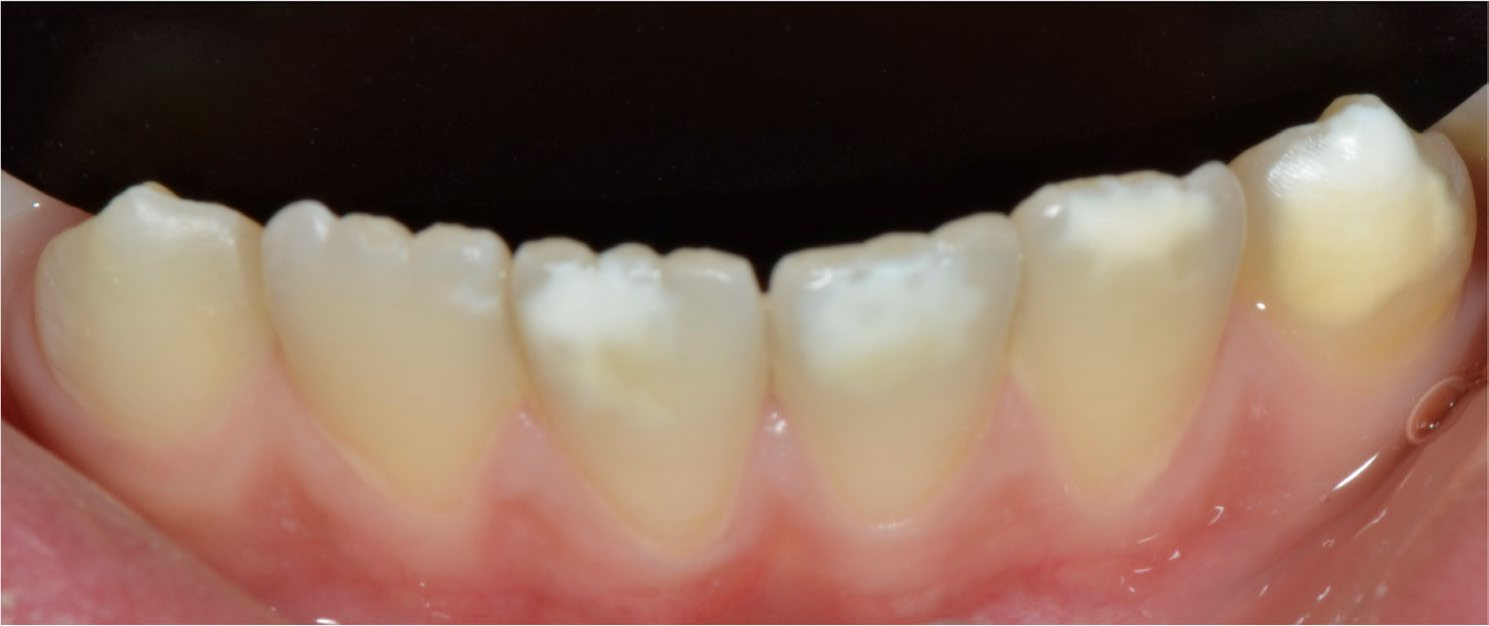
MIH-1: Limited opacities on mandibular incisors and canines

**Fig. 3:**
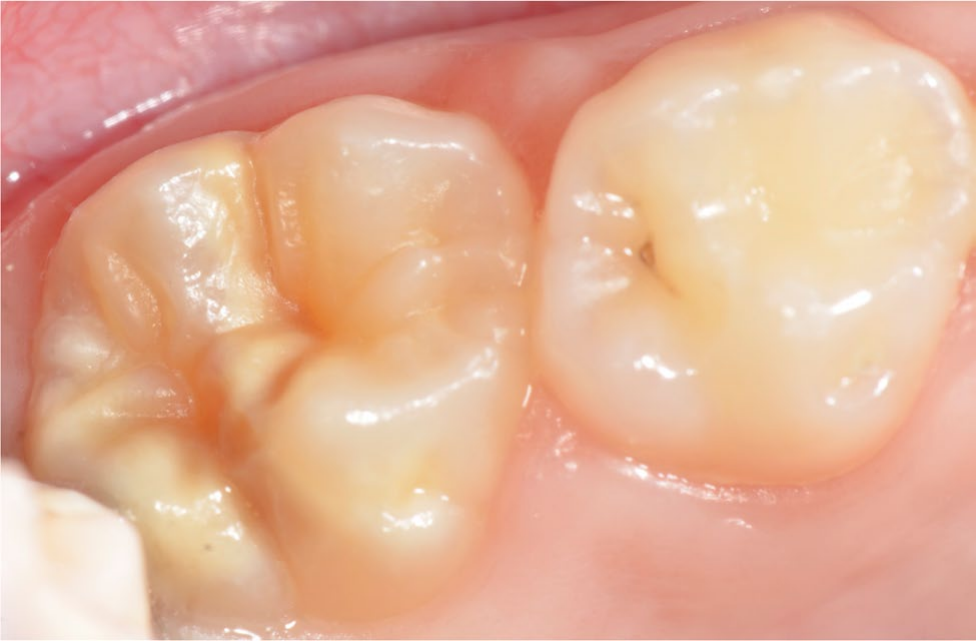
MIH-1: Limited opacities on a maxillary first permanent molar

**Fig. 4:**
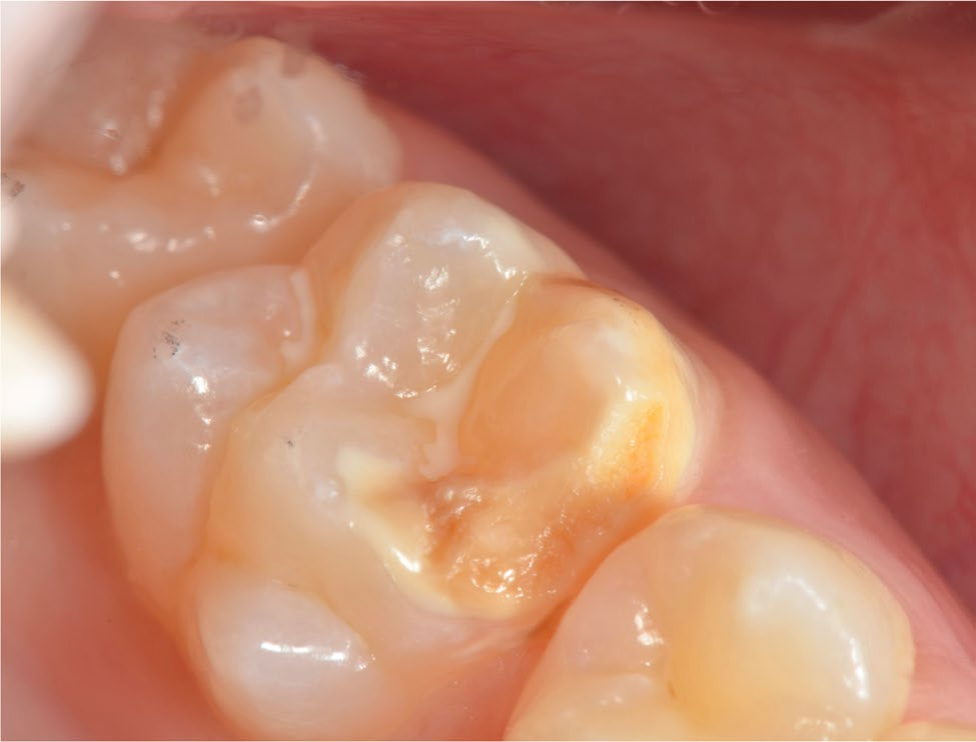
MIH-2: Post-eruptive enamel breakdown on maxillary first permanent molar

**Fig. 5:**
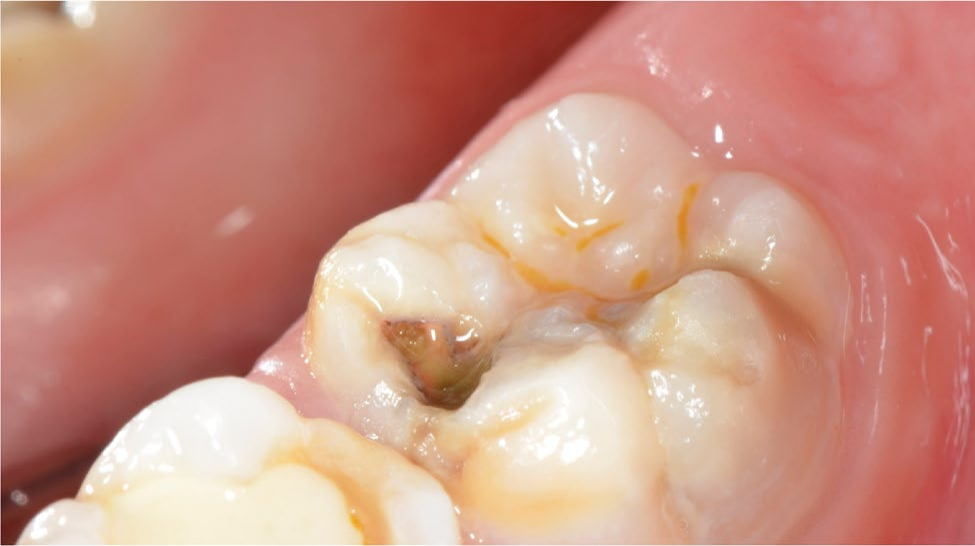
MIH-2: Post-eruptive enamel breakdown and caries on mandibulary first permanent molar

**Fig. 6:**
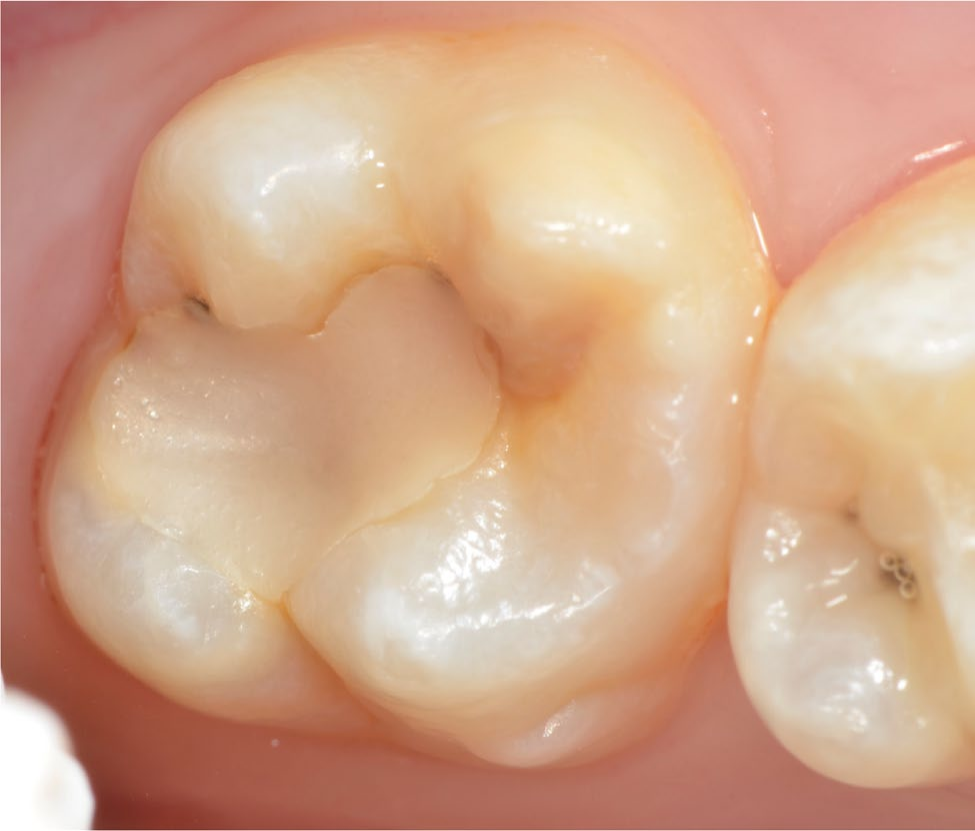
MIH-2: Atypical composite restoration on maxillary first permanent molar

The oral examinations of these 50 MIH-diagnosed patients were repeated at the Department of Pedodontics at Ege University Faculty of Dentistry to determine which teeth and how many teeth in total were affected by MIH, and the findings were recorded. Patients were categorized into MIH-1 and MIH-2 groups based on the severity criteria defined by Kılınç et al. [5]. (2019). Patients were classified as MIH-1 if they had well-defined opacities on one or more permanent first molars or incisors (Figs. 1, 2 and 3). Those who had at least one permanent first molar or incisor with post-eruptive enamel breakdown (Figs. 4 and 5), atypical restoration (Fig. 6), or a missing permanent first molar due to MIH were classified as MIH-2. All parents and children received oral hygiene education and were informed about MIH. The affected teeth were documented with photographs. As shown in Figs. 5 and 6, patients requiring treatment were referred to our clinic.

### Statistical analysis

The statistical analysis was performed using SPSS version 25.0 (IBM, New York, USA). Descriptive statistics were presented as frequency (n) and percentage (%) for categorical variables, median (min–max) for non-normally distributed data, and mean ± standard deviation for normally distributed variables. The Shapiro-Wilk test was used to evaluate the assumption of normality, and the Levene test was used to assess variance homogeneity.

### Dependent variables

Teeth affected by MIH: Categorized using FDI notation [12].

Severity:

- MIH-1: Limited opacities (Figs. 1, 2 and 3).
- MIH-2: Post-eruptive enamel breakdown, caries, atypical restorations, or extractions¹³’¹⁴ (Figs. 4, 5 and 6).

### Independent variables

*Sociodemographic Factors*: Age, gender, parental education, income, family type, employment, and smoking.

*Systemic Factors*: Respiratory conditions, atopic conditions, infections, neonatal jaundice, and antibiotic use before age 3.

*Prenatal/Perinatal Factors*: Pregnancy complications, delivery type, birth weight, birth length, gestational age, parity, and supplement use.

*Feeding*: Exclusive and total breastfeeding duration.

*Biochemical Parameters*: Categorized using reference ranges from Clinical Biochemistry Laboratory. A complete blood count was performed using Sysmex XN3100 analyzers. Iron levels, ferritin, total iron-binding capacity (TIBC), and creatinine were measured photometrically. Vitamin B12 and total IgE levels were analyzed using immunochemistry methods. The Westergren technique was used to measure the sedimentation rate.

For comparisons between two independent groups, normally distributed variables were analyzed using the Independent Samples T-test, while non-normally distributed variables were compared using the Mann-Whitney U test. The Pearson Chi-Square test was used to examine relationships between categorical variables when the expected value assumption (> 5) was met; otherwise, Fisher’s Exact test was applied.

## Results

### A) Case group and control group

Out of 700 examined pediatric patients, 84 were diagnosed with MIH, indicating a prevalence rate of 12%. Within the case group, 24% (*n* = 12) were classified as MIH-1 (mild opacities) (Figs. 1 and 2-3) and 76% (*n* = 38) as MIH-2 (severe defects) (Figs. 4 and 5-6). The average number of affected teeth was significantly higher in the MIH-2 group (8.71 ± 3.39) compared to the MIH-1 group (4.17 ± 2.98). The most frequently affected teeth were the maxillary right first molar (16) and the mandibular left first molar (36). Among the case group, 72% (*n* = 36) had both maxillary first molars affected, while 66% (*n* = 33) had both mandibular first molars affected. Regarding permanent incisors, the upper left central incisor (21) was the most affected. Additionally, 64% (*n* = 32) had both maxillary central incisors affected, 58% (*n* = 29) had both mandibular central incisors affected, and 24% (*n* = 12) had both maxillary lateral incisors affected. The mandibular right canine (43) showed the highest frequency of MIH among permanent canine. 8% (*n* = 4) having both mandibular canines affected, while 92% (*n* = 46) had no maxillary canines affected.

The study included 100 participants: Among 84 children diagnosed with MIH, 34 parents declined participation; thus, 50 children who diagnosed as MIH were included in the case group and 50 healthy controls for comparison analyses. The case group comprised 33 females (66%) and 17 males (34%), with a mean age of 9.18 ± 1.67 years. The control group included 26 females (52%) and 24 males (48%), with a mean age of 9.14 ± 2.08 years. Patients in the case group had a mean BMI of 17.52 ± 3.92. Among them, 80% were classified as normal weight, 4% as underweight (BMI ≤ −2 SDS), and 16% as obese (BMI ≥ + 2 SDS). In contrast, 98% of the control group had normal weight, with only 2% classified as obese. The majority (84%) of the case group lived in nuclear families, while 16% lived in extended families. When analyzing the distribution of MIH by gender, no significant difference was found between gender, age and MIH. However, a significant association was observed between economical status and the presence of defects. The prevalence of MIH was found to be higher in children from families with low economic status compared to those from families with normal or high economic status (*p* < 0.001). In parallel with this situation, there was a higher prevalence in the malnourished group as having MIH (*p* = 0.009). In the case group, 26% of mothers had primary education, and 74% were unemployed. Among fathers, 30% had primary education, and 96% were employed. Regarding birth-related factors, 54% (*n* = 27) of the case group were delivered via normal spontaneous vaginal delivery (NSVD), while 46% (*n* = 23) were born by cesarean section (*p* > 0.05). Prematurity (≤ 37 weeks) was more common in the case group (22%) than in the control group (12%). Birth prematurity, number of births, exposure to cigarette smoke, diarrhea frequency, upper airway system infections, urinary tract infections, antibiotic use and age were found to be significantly associated with MIH. Similarly, having had chickenpox, gastroenteritis hand and foot mouth disease were also linked with MIH etiology (*p* < 0.005). There were significant associations (*p* < 0.05) between patient groups and BMI, income level, parental education, maternal employment, birth weight, gestational age, number of births, household smoking, feeding method, respiratory diseases (tonsillitis, bronchitis, asthma), febrile conditions, allergic rhinitis, atopic dermatitis, urinary tract infections, neonatal jaundice, hand-foot-mouth disease, acute gastroenteritis, cardiac disease, age of illness onset, and antibiotic use. The case group had higher rates of borderline obesity, lower income, unemployed mothers, and parents with primary education. Birth weight ≥ 3.5 kg, gestational age < 37 weeks, and a higher number of maternal births were also more common in the case group. Household smoking and mixed feeding (breast milk and complementary foods) were more frequent. Respiratory diseases, febrile conditions, allergic rhinitis, atopic dermatitis, urinary tract infections, neonatal jaundice, acute gastroenteritis, and cardiac disease were predominantly observed in the case group. Early illness onset and antibiotic use before age three were also more common.

Regarding breastfeeding, 58% (*n* = 29) of the case group were exclusively breastfed for 6 months, while 12% (*n* = 6) received breast milk with formula and 12% (*n* = 6) with complementary feeding. The onset of illness occurred before the age of 3 in 82% of cases. Among 49 respondents, 91.8% used antibiotics before the age of 3, while 8.2% used them only after the age of 3. Statistical analysis revealed a significant association (*p* < 0.05) between the presence of MIH, antibiotic use, and the age at onset of illnesses before 3 years. Comparison of demographic and clinical characteristics among patient groups and control groups, and the relationships between these characteristics were shown in Table 1. A significant difference was found in the average breastfeeding period between children with and without MIH. Table 2 indicates that children exclusively breastfed during the first six months exhibit a higher prevalence of molar incisor hypomineralization (MIH) compared to their non-exclusively breastfed counterparts.

**Table 1:** Comparison of demographic and clinical characteristics among patient groups and the relationships between these characteristics

		Study Group			Control Group				
		*n*	%	%G.	*n*	%	%G.	Test Statistics	*p*
**Gender**	**Girl**	33	57,9	66,0	24	42,1	48,0	3,305	0,069
	**Boy**	17	39,5	34,0	26	60,5	52,0		
**Age (years)**	**6–12**	50			50			−0,319	0.750
**Family type**	**Extended**	8	66,7	16,0	4	33,3	8,0	1,515	0,218
	**Nuclear**	42	47,7	84,0	46	52,3	92,0		
**BMI**	**Developmental Delay**	2	100,0	4,0	0	0,0	0,0	7,963‡	0,009*
	**Normal**	40	44,9	80,0	49	55,1	98,0		
	**Obesity**	8	88,9	16,0	1	11,1	2,0		
**Economical statue**	**Below minimum wage**	2	66,7	4,0	1	33,3	2,0	17,932‡	< 0,001*
	**Minimum wage**	18	90,0	36,0	2	10,0	4,0		
	**Above minimum wage**	30	39,0	60,0	47	61,0	94,0		
**Mother’s education**	**Primary ed.**	13	68,4	26,0	6	31,6	12,0	8,674	0,013*
	**Secondary ed.**	24	58,5	48,0	17	41,5	34,0		
	**Higher ed.**	13	32,5	26,0	27	67,5	54,0		
**Father’ education**	**Primary ed.**	15	78,9	30,0	4	21,1	8,2	16,945	< 0,001*
	**Secondary ed.**	23	62,2	46,0	14	37,8	28,6		
	**Higher ed.**	12	27,9	24,0	31	72,1	63,3		
**Employment statue of mother**	**Unemployed**	37	71,2	74,0	15	28,8	30,0	19,391	< 0,001*
	**Employed**	13	27,1	26,0	35	72,9	70,0		
**Employment statue of father**	**Unemployed**	2	100,0	4,0	0	0,0	0,0	-‡	0,495
	**Employed**	48	49,5	96,0	49	50,5	100,0		
**Delivery type**	**NSVD**	27	57,4	54,0	20	42,6	40,0	1,967	0,161
	**CS**	23	43,4	46,0	30	56,6	60,0		
**Pregnancy complications**	**Yes**	11	68,8	22,0	5	31,3	10,0	2,679	0,102
	**No**	39	46,4	78,0	45	53,6	90,0		
**Birth weight**	**≤ 2**,**5 kg LBW**	0	0,0	0,0	5	100,0	10,0	15,225‡	< 0,001*
	**2**,**5 kg- 3**,**5 kg-NBW**	23	39,7	46,0	35	60,3	70,0		
	**≥ 3**,**5 kg-LGA**	27	73,0	54,0	10	27,0	20,0		
**Birth lenght**	**≤ 50 cm**	26	45,6	52,0	31	54,4	62,0	1,020	0,313
	**> 50 cm**	24	55,8	48,0	19	44,2	38,0		
**Gestational agé**	**≤ 37-premature**	11	64,7	22,0	6	35,3	12,0	8,113	0,017*
	**37–40**	14	33,3	28,0	28	66,7	56,0		
	**≥ 40**	25	61,0	50,0	16	39,0	32,0		
**Number of births**	**1**	6	22,2	12,2	21	77,8	42,0	19,334‡	< 0,001*
	**2**	22	51,2	44,9	21	48,8	42,0		
	**3**	16	80,0	32,7	4	20,0	8,0		
	**4**	3	60,0	6,1	2	40,0	4,0		
	**5**	2	100,0	4,1	0	0,0	0,0		
	**6**	0	0,0	0,0	2	100,0	4,0		
**Exposure to sigarette smoke**	**Yes**	24	77,4	48,0	7	22,6	14,0	13,511‡	< 0,001*
	**No**	26	37,7	52,0	43	62,3	86,0		
**Vit D supplement**	**Yes**	4	28,6	8,0	10	71,4	20,0	2,990	0,084
	**No**	46	53,5	92,0	40	46,5	80,0		
**Iron supplement**	**Yes**	1	50,0	2,0	1	50,0	2,0	-‡	1,000
	**No**	49	50,0	98,0	49	50,0	98,0		
**Multivitamin**	**Yes**	3	25,0	6,0	9	75,0	18,0	3,409	0,065
	**No**	47	53,4	94,0	41	46,6	82,0		
**Feeding**	**Exclusive breastfeeding**	0	0,0	0,0	6	100,0	12,0	6,758‡	0,026*
	**Breastfeeding and formula**	48	52,7	96,0	43	47,3	86,0		
	**Formula and complementery feeding**	2	66,7	4,0	1	33,3	2,0		
**Tonsillitis**	**Yes**	27	93,1	54,0	2	6,9	4,0	30,355	< 0,001*
	**No**	23	32,4	46,0	48	67,6	96,0		
**Bronchitis**	**Yes**	13	76,5	26,0	4	23,5	8,0	5,741	0,017*
	**No**	37	44,6	74,0	46	55,4	92,0		
**Asthma**	**Yes**	9	90,0	18,0	1	10,0	2,0	7,111	0,008*
	**No**	41	45,6	82,0	49	54,4	98,0		
**Otitis**	**Yes**	9	75,0	18,0	3	25,0	6,0	3,409	0,065
	**No**	41	46,6	82,0	47	53,4	94,0		
**Febrile conditions**	**Yes**	16	84,2	32,0	3	15,8	6,0	10,981	0,001*
	**No**	34	42,0	68,0	47	58,0	94,0		
**Allergic rhinitis**	**Yes**	17	81,0	34,0	4	19,0	8,0	10,187	0,001*
	**No**	33	41,8	66,0	46	58,2	92,0		
**Atopic dermatitis**	**Yes**	11	91,7	22,0	1	8,3	2,0	9,470	0,002*
	**No**	39	44,3	78,0	49	55,7	98,0		
**Food allergy**	**Yes**	8	61,5	16,0	5	38,5	10,0	0,796	0,372
	**No**	42	48,3	84,0	45	51,7	90,0		
**General allergies**	**Yes**	14	60,9	28,0	9	39,1	18,0	1,412	0,235
	**No**	36	46,8	72,0	41	53,2	82,0		
**Sepsis**	**Yes**	4	100,0	8,0	0	0,0	0,0	-‡	0,117
	**No**	46	47,9	92,0	50	52,1	100,0		
**UTI**	**Yes**	20	87,0	40,0	3	13,0	6,0	16,318	< 0,001*
	**No**	30	39,0	60,0	47	61,0	94,0		
**ID**	**Yes**	1	100,0	2,0	0	0,0	0,0	-‡	1,000
	**No**	49	49,5	98,0	50	50,5	100,0		
**Neonatal Jaundice**	**Yes**	20	71,4	40,0	8	28,6	16,0	7,143	0,008*
	**No**	30	41,7	60,0	42	58,3	84,0		
**Developmental Delay**	**Yes**	2	100,0	4,0	0	0,0	0,0	-‡	0,495
	**No**	48	49,0	96,0	50	51,0	100,0		
**Immunodeficiency**	**Yes**	1	100,0	2,0	0	0,0	0,0	-‡	1,000
	**No**	49	49,5	98,0	50	50,5	100,0		
**Measles**	**Yes**	5	100,0	10,0	0	0,0	0,0	-‡	0,056
	**No**	45	47,4	90,0	50	52,6	100,0		
**Chickenpox**	**Yes**	6	75,0	12,0	2	25,0	4,0	-‡	0,269
	**No**	44	47,8	88,0	48	52,2	96,0		
**Hand Foot Mouth Disease**	**Yes**	10	76,9	20,0	3	23,1	6,0	4,332	0,037*
	**No**	40	46,0	80,0	47	54,0	94,0		
**AGE**	**Yes**	6	100,0	12,0	0	0,0	0,0	-‡	0,027*
	**No**	44	46,8	88,0	50	53,2	100,0		
**Cardiac Diseases**	**Yes**	7	100,0	14,0	0	0,0	0,0	-‡	0,012*
	**No**	43	46,2	86,0	50	53,8	100,0		
**Pneumonia**	**Yes**	5	100,0	10,0	0	0,0	0,0	-‡	0,056
	**No**	45	47,4	90,0	50	52,6	100,0		
**Age at Onset Illnesses**	**No illnesses**	0	0,0	0,0	16	100,0	32,0	41,661	< 0,001*
	**had < 3 age**	41	80,4	82,0	10	19,6	20,0		
	**had > 3 age**	9	27,3	18,0	24	72,7	48,0		
**Antibiotic Use before age 3**	**Yes**	45	81,8	91,8	10	18,2	20,0	51,722	< 0,001*
	**No**	4	9,1	8,2	40	90,9	80,0		

*Significant values

‡: Pearson Chi Square test, %: Row percentage and %G.: Column percentage for patient groups

*LBW* Low Birth Weight, *NBW* Normal Birth Weight, *LGA* Large for Gestational Age

*AGE* Acute Gastroenteritis, *ID* Intellectuel Disability, *UTI* Urine Tract Infection

**Table 2:** Comparison of anthropometric measurements and feeding characteristics between the study group and the control group

	Study group		Control group			
	**Min.-Max.**	**Mean** ± **standard deviation**	**Min.-max.**	**Mean** ± **standard deviation**	**Test Statistics**	***p***
**Duration of exclusive breastfeeding (months)**	0–10	5,02 ± 2,4(6)	0–14	6,44 ± 2,52(6)	−2,329	0,020*
**Total duration of breastfeeding ((months)**	0–36	18,72 ± 9,48(20)	0–30	15,74 ± 7,81(15)	−1,599	0,110

*Significant value

[5,02 ± 2,4 vs. 6,44 ± 2,52] (*p* < 0.05) (Table 2).

### B) MIH-1 and MIH-2 groups

Among 50 MIH-positive patients, when teeth affected by MIH were categorized as MIH-1 and MIH-2 using the FDI notation, no significant differences were found between the groups in terms of BMI, mode of delivery, birth weight, economic status, maternal education, and feeding patterns before the age of 1 (Table 3). No significant associations were found with gender, family type, father’s employment, cesarean section, pregnancy complications, birth length, vitamin supplementation, otitis, food allergies, sepsis, intellectual disability, measles, chickenpox, or pneumonia (*p* > 0.05). A statistically significant difference (*p* < 0.05) was observed in exclusive breastfeeding duration between the case and control groups, with the control group having a longer breastfeeding duration. No significant differences were found in age, maternal age, paternal age, or total breastfeeding duration (*p* > 0.05).

**Table 3:** Distribution of demographic characteristics according to severity groups and the relationships between them

		MIH-1			MIH-2				
		*n*	%	%G.	*n*	%	%G.	Test statistics	*p*
**Gender**	Girl	9	27,3	75,0	24	72,7	63,2	-	0,510
	Boy	3	17,6	25,0	14	82,4	36,8		
**BMI**	≤−2-patological Developmental delay	0	0,0	0,0	2	100,0	5,3	0,941	0,804
	−2/+2 ı-normal	11	27,5	91,7	29	72,5	76,3		
	≥ 2-patological, Obesity	1	12,5	8,3	7	87,5	18,4		
**Delivery type**	NSVD	7	25,9	58,3	20	74,1	52,6	0,119‡	0,730
	C-S	5	21,7	41,7	18	78,3	47,4		
**Pregnancy Complications**	Yes	3	27,3	25,0	8	72,7	21,1	-	1,000
	No	9	23,1	75,0	30	76,9	78,9		
**Birth Weight**	2,5 kg- 3,5 kg-Normal	5	21,7	41,7	18	78,3	47,4	0,119‡	0,730
	≥ 3,5 kg-LGA	7	25,9	58,3	20	74,1	52,6		
**Birth lenght**	≤ 50 cm	7	26,9	58,3	19	73,1	50,0	0,254‡	0,614
	> 50 cm	5	20,8	41,7	19	79,2	50,0		
**Gestational age**	≤ 37-premature	2	18,2	16,7	9	81,8	23,7	1,449	0,568
	37–40	5	35,7	41,7	9	64,3	23,7		
	≥ 40	5	20,0	41,7	20	80,0	52,6		
**Number of births**	1	3	50,0	25,0	3	50,0	8,1	4,136	0,329
	2	6	27,3	50,0	16	72,7	43,2		
	3	2	12,5	16,7	14	87,5	37,8		
	4	1	33,3	8,3	2	66,7	5,4		
	5	0	0,0	0,0	2	100,0	5,4		
**Family type**	Extended	1	12,5	8,3	7	87,5	18,4	-	0,661
	Nuclear	11	26,2	91,7	31	73,8	81,6		
**Economical statue**	Below minimum wage	0	0,0	0,0	2	100,0	5,3	0,482	1,000
	Minimum wage	4	22,2	33,3	14	77,8	36,8		
	Above minimum wage	8	26,7	66,7	22	73,3	57,9		
**Mother’s education**	Primary ed.	1	7,7	8,3	12	92,3	31,6	2,593	0,299
	Secondary ed.	7	29,2	58,3	17	70,8	44,7		
	Higher ed.	4	30,8	33,3	9	69,2	23,7		
**Father‘s education**	Primary ed	2	13,3	16,7	13	86,7	34,2	2,851	0,252
	Secondary ed	5	21,7	41,7	18	78,3	47,4		
	Higher ed	5	41,7	41,7	7	58,3	18,4		
**Employment statue of mother**	Unemployed	7	18,9	58,3	30	81,1	78,9	-	0,256
	Employed	5	38,5	41,7	8	61,5	21,1		
**Employment statue of father**	Unemployed	1	50,0	8,3	1	50,0	2,6	-	0,426
	Employed	11	22,9	91,7	37	77,1	97,4		
**Exposure to sigarette smoke**	Yes	7	29,2	58,3	17	70,8	44,7	0,675‡	0,411
	No	5	19,2	41,7	21	80,8	55,3		
**Vitamin D supplement**	Yes	1	25,0	8,3	3	75,0	7,9	-	1,000
	No	11	23,9	91,7	35	76,1	92,1		
**Iron supplement**	Yes	1	100,0	8,3	0	0,0	0,0	-	0,240
	No	11	22,4	91,7	38	77,6	100,0		
**Multivitamin**	Yes	1	33,3	8,3	2	66,7	5,3	-	1,000
	No	11	23,4	91,7	36	76,6	94,7		
**< 1 age feeding**	Breastfeeding and complementary feeding	12	25,0	100,0	36	75,0	94,7	-	1,000
	Only formula	0	0,0	0,0	2	100,0	5,3		

When the biochemical findings were compared between MIH-1 and MIH-2 patients, significant differences were observed in urea, total iron-binding capacity (TIBC), lymphocyte, monocyte, and eosinophil levels (*p* < 0.05), with the MIH-2 group exhibiting higher lymphocyte, monocyte, and eosinophil levels. However, no significant differences were found between the groups in terms of ALP, creatinine, calcium, phosphorus, iron, leukocytes, neutrophils, basophils, erythrocytes, hemoglobin, hematocrit, MCV, MCH, MCHC, platelets, erythrocyte sedimentation rate, total IgE, ferritin, vitamin B12, PTH, and vitamin D levels (*p* > 0.05) (Table 4).

**Table 4:** Distribution and comparison of biochemical measurements according to severity groups

	MIH-1 (*n* = 12)		MIH-2 (*n* = 38)			
	Median (Min.-Maks.)	Mean.±Standard Deviation.	Median (Min.-Maks.)	Mean.±Standard Devitation.	Test Statistics	*p*
**ALP (U/L)**	233 (131–397)	248,92 ± 82,14	247,5 (152–397)	255,84 ± 59,73	−0,319	0,751
**Urine (mg/dL)**	22,5 (14–31)	21,92 ± 4,81	18,5 (12–28)	19,08 ± 3,91	2,071	0,044*
**Creatinine (mg/dL)**	0,44 (0,3 − 0,56)	0,44 ± 0,08	0,42 (0,28 − 0,69)	0,44 ± 0,09	−0,649†	0,517
**Ca (mg/dL)**	9,8 (9,1–10,4)	9,82 ± 0,35	9,65 (8,9–10,2)	9,63 ± 0,33	1,689	0,098
**P (mg/dL)**	4,71 (3,89 − 5,55)	4,64 ± 0,51	4,55 (3,58 − 6,65)	4,57 ± 0,6	−0,613†	0,540
**Fe (µg/dL)**	73 (28–130)	68,83 ± 30,81	(75,5) 24–135	75,32 ± 26,33	−0,714	0,479
**TIBC (µg/dL)**	393 (265–479)	389,25 ± 54,03	(358,5) 252–440	357,03 ± 44,11	2,090	0,042*
**Leucosyte (10^3/µL)**	6,8 (4,1–9,2)	6,71 ± 1,46	7,19 (5–15,29)	8,11 ± 2,72	−1,567†	0,117
**Neutrophyl # (10^3/µL)**	3,7 (1,74 − 6,32)	3,84 ± 1,34	3,87 (2,09–9,49)	4,19 ± 1,96	−0,159†	0,874
**Lymphocyte# (10^3/µL)**	2,15 (1,03–3,41)	2,22 ± 0,64	2,82 (1,22 − 7,37)	2,97 ± 1,07	−2,499†	0,012*
**Monocyte# (10^3/µL)**	0,5 (0,27 − 0,61)	0,48 ± 0,09	0,57 (0,34 − 1,25)	0,62 ± 0,22	−2,195†	0,028*
**Eosinophil # (10^3/µL**	0,11 (0,06 − 0,44)	0,14 ± 0,11	0,23 (0,06 − 1,5)	0,29 ± 0,26	−2,594†	0,009*
**Basophile #(10^3/µL)**	0,04 (0,02 − 0,06)	0,04 ± 0,02	0,04 (0,01 − 0,14)	0,04 ± 0,03	−0,715†	0,474
**Eritrocyte (10^6/µL**	4,68 (4,32 − 5,08)	4,7 ± 0,28	4,84 (4,14 − 5,51)	4,8 ± 0,3	−0,996	0,324
**Hg (g/dL)**	12,95 (11,1–13,8)	12,92 ± 0,82	13,2 (11,1–14,5)	13,12 ± 0,77	−0,791	0,433
**HCT (%)**	38,15 (33,7–41,5)	38,25 ± 2,37	39 (33,6–42,5)	38,84 ± 2	−0,848	0,400
**MCV (f/L)**	82 (76,6–85,3)	81,42 ± 2,78	81 (75,7–87,8)	81,09 ± 3,38	0,301	0,765
**MCH (pg)**	27,8 (24,2–29,6)	27,53 ± 1,45	27,65 (23,5–30,3)	27,39 ± 1,51	0,279	0,782
**MCHC (g/dL)**	33,65 (31,6–36,5)	33,78 ± 1,17	33,8 (30,8–36,5)	33,78 ± 1,17(33,8)	−0,003	0,997
**PLT (Trombocyte) (**	335 (234–388)	327,75 ± 48,38	308,5 (187–479)	320,21 ± 75,72(308,5)	0,405	0,688
**Sedimentation 1 h (mm)**	5 (2–24)	8,58 ± 6,44	7 (2–35)	7,92 ± 6,62	−0,230†	0,818
**Total IgE (kU/L)**	28,5 (10,6–189)	46,6 ± 49,44	69,7 (0,8–1389)	164,63 ± 268,98	−1,090†	0,276
**Ferritin (µg/L)**	23,45 (10,4–71,8)	29,86 ± 18,43	30,5 (10,9–115)	35,68 ± 22,79	−0,807†	0,420
**Vitamin B12 (ng/L)**	365 (224–726)	396,25 ± 142,18	396,5 (192–873)	414,37 ± 159,74	−0,125†	0,901
**PTH (ng/L)**	42,35 (20,7–92,1)	46,12 ± 21,13	30,75 (19,7–185)	42,77 ± 31,25	−1,113†	0,266
**Vitamin D (ng/mL)**	16,5 (7–22)	16,17 ± 4,61	17,5 (7–34)	17,29 ± 6,61	−0,341†	0,733

## Discussion

Molar Incisor Hypomineralization (MIH) is an increasingly recognized global dental health concern, characterized by enamel defects that lead to dental hypersensitivity, rapid caries progression, masticatory dysfunction, and aesthetic concerns. The multifactorial etiology of MIH remains a subject of ongoing investigation, with genetic, systemic, and environmental factors playing potential roles [10, 13].

Our study, which examined MIH prevalence and etiological risk factors among patients attending the Ege University Faculty of Dentistry, found an MIH prevalence rate of 12%, aligning with previous studies conducted in Turkey [5–7]. Consistent with the literature, the maxillary first molars and central incisors were the most affected teeth, while mandibular molars were less frequently involved [6, 14].

Several studies have highlighted the role of socioeconomic factors in MIH prevalence, suggesting that lower socioeconomic status is associated with a higher risk of MIH [15]. In our study, significant associations were found between MIH prevalence and lower parental education levels, maternal unemployment, higher birth order, and household smoking exposure. These findings align with existing research indicating that limited access to healthcare, inadequate oral hygiene practices, and poor nutrition may contribute to MIH development.

Systemic and perinatal factors have also been linked to MIH. Our study found a significant association between preterm birth and MIH, consistent with findings from Silva et al. [13] and Wu et al. [16] which suggest that premature birth may disrupt enamel mineralization during critical developmental phases. Additionally, early childhood illnesses, particularly respiratory infections, high fever episodes, and gastrointestinal disorders, were significantly associated with MIH, supporting prior evidence of their impact on ameloblast function [1, 17–19].

The role of breastfeeding in MIH remains controversial. While some studies suggest that prolonged breastfeeding may have a protective effect [20], others indicate a potential risk for enamel defects [21]. Our study found that the duration of exclusive breastfeeding was significantly longer in the healthy control group, suggesting a possible protective effect against MIH. However, further research with more cases is needed to clarify the underlying mechanisms.

The relationship between MIH and vitamin and mineral deficiencies has been debated. Our study did not find significant associations between MIH severity and serum levels of calcium, phosphorus, parathyroid hormone (PTH), vitamin B12, or hemoglobin. Although urea, total iron-binding capacity, and lymphocyte levels were slightly elevated, the differences were statistically significant but not clinically meaningful.

However, increased total iron-binding capacity (TIBC) levels in mild MIH cases and elevated eosinophil and monocyte levels in severe cases suggest a potential role of systemic inflammation [22]. Mild monocytosis observed in patients with MIH2 was interpreted as a response to bone marrow inflammation, given the higher inflammatory burden in this group. Additionally, eosinophil counts were numerically higher in MIH2, which was attributed to the greater prevalence of atopic conditions in these patients. These findings warrant further investigation into the inflammatory aspects of MIH pathogenesis.

While previous research, including a systematic review and meta-analysis by Hujoel et al., has suggested that vitamin D supplementation in early life may be associated with reduced caries risk, our study did not find a statistically significant association between MIH and the use of vitamin D or multivitamin supplements [23]. Notably, although the number of individuals receiving vitamin D supplementation was higher in the healthy control group compared to those diagnosed with MIH, this difference was not statistically significant. These findings suggest that, within the limitations of our sample, vitamin D supplementation alone may not be a protective factor against MIH development.

The results of this study were consistent with those of other studies in the literature and there was no statistically significant relationship between the vitamin D levels of the severity groups. However, vitamin D levels of the patients in the case group were significantly and mildly moderately low (median = 17 ng/mL).

This is particularly relevant given that studies from Türkiye report a high prevalence of vitamin D deficiency among children, with rates approaching 40% nationally [24, 25] and similar deficiency patterns also noted in paediatric populations in the Aegean region [25]. Considering these data, the absence of a significant association in our cross-sectional analysis does not exclude a role for early-life vitamin D deficiency in MIH development. Longitudinal studies, tracking vitamin D status from birth through the tooth development period, are required to clarify this potential relationship.

Genetic susceptibility has been increasingly explored in MIH research. Bussaneli et al. [26] identified associations between TGFBR1 gene polymorphisms and severe MIH cases, highlighting potential genetic contributions. Moreover, studies have demonstrated links between MIH and allergic conditions such as atopic dermatitis and allergic rhinitis [20, 27] findings that are consistent with our study results.

Finally, the potential role of antibiotic exposure in MIH development has been well-documented. Our study found a significant association between antibiotic use in the first three years of life and MIH, corroborating previous findings that early-life antibiotic exposure may alter enamel formation [13, 28] While this study identifies significant associations, further research is required to determine causal relationships between these factors and MIH development.

In conclusion, our findings support the hypothesis that MIH etiology is multifactorial, with significant contributions from socioeconomic, systemic, perinatal, and genetic factors. Future research should focus on large-scale longitudinal and genetic studies to elucidate the multifactorial etiology of MIH and identify potential preventive strategies.

In this study, children with more severe MIH (MIH-2 group) exhibited higher lymphocyte, monocyte, and eosinophil counts, as well as higher urea and total iron-binding capacity (TIBC) levels, compared to controls. While these findings are intriguing, the biological mechanisms underlying these associations are not fully understood. Elevated lymphocyte and eosinophil levels may reflect underlying inflammatory or allergic conditions, which have been speculated to influence enamel development. Higher TIBC may indicate altered iron metabolism; however, the clinical significance of this finding in the context of MIH remains unclear. Previous studies have not consistently reported such haematological associations with MIH, and the cross-sectional design of our study precludes establishing causality. Further longitudinal studies with larger sample sizes are needed to explore whether these haematological differences are coincidental or related to MIH pathogenesis.

### Limitations

This study has some limitations. The study utilised a convenience sampling approach without randomisation, which may limit the generalisability of the findings. Although the clinic receives a diverse patient population, the sample may not fully represent the general paediatric population.

Although the age range was set between 6 and 12 years for practical and clinical reasons, we acknowledge that the age of 8 years is considered ideal for MIH diagnosis. Therefore, this broader age range may have introduced variability in diagnostic accuracy.

The relatively small case-control sample size may have reduced the ability to detect weaker associations. Reliance on parental recall for early-life health data could introduce bias, despite encouraging the use of written health records. Furthermore, blood parameters measured at the time of examination may not accurately reflect the child’s status during enamel formation, limiting causal interpretation. In particular, vitamin D levels during early developmental stages were not assessed; although deficiency was common among affected children, supplementation history and current levels did not show a significant association with MIH.

## Conclusion

MIH was found to be relatively common in this paediatric population in Izmir, Türkiye.

Several factors, including respiratory diseases, atopic conditions, early antibiotic exposure, and shorter exclusive breastfeeding duration, were significantly associated with its occurrence. Although serum vitamin D levels were measured at the time of this study, these values may not accurately reflect the child’s vitamin D status during the critical period of enamel formation in early childhood, which may have limited our ability to detect a potential association.

These findings suggest the potential importance of early diagnosis, promotion of exclusive breastfeeding during the first six months, and preventive dental strategies such as fluoride application and fissure sealants. A multidisciplinary approach; engaging pediatricians, allergists, and pediatric dentists is essential to address modifiable risk factors and improve outcomes. Further large-scale, longitudinal studies are needed to clarify the complex etiology of MIH and guide effective prevention programs.

## Supplementary Information

Supplementary Material 1

## Data Availability

The datasets used and analyzed during the current study are available from the corresponding author on reasonable request.

## Abbreviations

MIH: Molar Incisor Hypomineralization
BMI: Body Mess Index
SDS: Standardized Deviation Score
ALP: Alkaline Phosphatase
TIBC: Total Iron Binding Capacity
