# Supplementary material for: Prevalence and associated factors of molar incisor hypomineralization in children: a cross-sectional study
Source: BMC Oral Health. 2025 Nov 25;25:1835. doi: 10.1186/s12903-025-07071-2 (PMC12648818; doi:10.1186/s12903-025-07071-2)
Supplement: Supplementary file 1 — Supplementary Material 1 [file 12903_2025_7071_MOESM1_ESM.docx]

# QUESTIONNAIRE

**Study Title:**

**Investigation of the Prevalence and Etiology of Molar-Incisor Hypomineralization in Childhood**

**For children aged 6–12 years**

Protocol No.: ....................

Case No.: ....................

File No.: ....................

MIH Severity Classification ( For the control group) : ☐ MIH-1 ☐ MIH-2

1. Date of Birth (day/month/year)

2. Mode of delivery (normal birth / cesarean)

3. Child’s age (months)

4. Were there any complications during pregnancy? ☐ Yes ☐ No

5. Gender ☐ Male ☐ Female

6. Birth weight (g)

7. Birth length (cm)

8. Gestational age (weeks)

9. Number of previous births by the mother and general nutritional habits during pregnancy

10. Family type ☐ Nuclear ☐ Extended

11. Total monthly family income ☐ Minimum wage ☐ Below minimum wage ☐ Above minimum wage

12. Mother’s age

13. Father’s age

14. Mother’s education level ☐ Primary education ☐ Secondary education ☐ Higher education

15. Father’s education level ☐ Primary education ☐ Secondary education ☐ Higher education

16. Mother’s employment status ☐ Housewife ☐ Employed

17. Father’s employment status ☐ Unemployed ☐ Employed

18. Is smoking allowed inside the house? ☐ Yes ☐ No

19. Is the child currently taking vitamin D supplements? ☐ Yes ☐ No

20. Is the child currently taking iron supplements? ☐ Yes ☐ No

21. Is the child currently taking multivitamin supplements? ☐ Yes ☐ No

22. Feeding method until the age of 1 ☐ Exclusive breastfeeding ☐ Breastfeeding + formula/complementary food ☐ Formula only ☐ Other

23. Duration of exclusive breastfeeding (months) *(For children who stopped breastfeeding or started complementary feeding)*

24. Total duration of breastfeeding (months) *(For children who stopped breastfeeding)*

25. Illnesses experienced before the age of 3 ☐ Bronchitis ☐ Asthma ☐ Chickenpox ☐ Otitis (ear infection) ☐ Tonsillitis ☐ Measles ☐ Food allergy ☐ Atopic eczema ☐ Rickets ☐ Sepsis ☐ Antibiotic use before age 3 ☐ Allergic rhinitis ☐ Pneumonia ☐ Acute gastroenteritis ☐ Febrile episodes (>38°C) ☐ Urinary tract infection ☐ Cardiac disease ☐ Intellectual disability ☐ Neonatal jaundice ☐ Hand-foot-mouth disease

26. At what age did these illnesses occur? *(Important: must be before 3 years of age)*

27. Allergens suspected in allergic conditions *(e.g. food, milk, pollen, dermatophytes, gluten, etc.)*

28. Weight (kg)

29. Height (cm)

30. Body Mass Index (BMI) Z-score
